# The effects of facemasks on airway inflammation and endothelial dysfunction in healthy young adults: a double-blind, randomized, controlled crossover study

**DOI:** 10.1186/s12989-018-0266-0

**Published:** 2018-07-04

**Authors:** Tianjia Guan, Songhe Hu, Yiqun Han, Ruoyu Wang, Qindan Zhu, Yaoqian Hu, Hanqing Fan, Tong Zhu

**Affiliations:** 1 BIC-EAST and SKL-ESPC, College of Environmental Sciences and Engineering and Centre for Environment and Health, Peking University, 5 Yiheyuan Road, Beijing, 100871 China; 2 School of Public Health, Chinese Academy of Medical Sciences and Peking Union Medical College, Beijing, 100730 China

**Keywords:** N95 facemask, Filtration efficiency, Ambient particulate matter, Airway inflammation, Endothelial dysfunction, Oxidative stress

## Abstract

**Background:**

Facemasks are increasingly worn during air pollution episodes in China, but their protective effects are poorly understood. We aimed to evaluate the filtration efficiencies of N95 facemasks and the cardiopulmonary benefits associated with wearing facemasks during episodes of pollution.

**Results:**

We measured the filtration efficiencies of particles in ambient air of six types of N95 facemasks with a manikin headform. The most effective one was used in a double-blind, randomized, controlled crossover study, involving 15 healthy young adults, conducted during 2 days of severe pollution in Beijing, China. Subjects were asked to walk along a busy-traffic road for 2 h wearing authentic or sham N95 facemasks. Clinical tests were performed four times to determine changes in the levels of biomarkers of airway inflammation, endothelial dysfunction, and oxidative stress within 24 h after exposure.

The facemasks removed 48–75% of number concentrations of ambient air particles between 5.6 and 560 nm in diameter. After adjustments for multiple comparison, the exhaled nitric oxide level and the levels of interleukin-1α, interleukin-1β, and interleukin-6 in exhaled breath condensate increased significantly in all subjects; however, the increases in those wearing authentic facemasks were statistically significantly lower than in the sham group. No significant between-group difference was evident in the urinary creatinine-corrected malondialdehyde level. In arterial stiffness indicators, the ejection duration of subjects wearing authentic facemasks was higher after exposure compared to the sham group; no significant between-group difference was found in augmentation pressure or the augmentation index.

**Conclusions:**

In young healthy adults, N95 facemasks partially reduced acute particle-associated airway inflammation, but neither systemic oxidative stress nor endothelial dysfunction improved significantly. The clinical significance of these findings long-term remains to be determined.

**Trial registration:**

The trial registration number (TRN) for this study is ChiCTR1800016099, which was retrospectively registered on May 11, 2018.

**Electronic supplementary material:**

The online version of this article (10.1186/s12989-018-0266-0) contains supplementary material, which is available to authorized users.

## Background

Exposure to particulate matter (PM) of aerodynamic diameter less than 2.5 μm (PM_2.5_) is the fourth leading health risk factor in China and is linked to more than 1.3 million premature deaths annually [1–3]. PM air pollution is associated with substantial increases in respiratory and cardiovascular morbidity and mortality, and has aroused great public concern in China, especially in highly polluted areas such as Beijing [4–6]. To reduce individual exposure, the use of facemasks, especially those trapping PM_2.5_, has become very popular, particularly since many cities experienced severe episodes of air pollution in 2011. N95 facemasks, which are generally recommended by health researchers, are the most widely used [7, 8].

The U.S. Food and Drug Administration (FDA) has explained that the N95 descriptor means that, on careful testing, the filter blocks at least 95% of very small (0.3-μm diameter) test particles. The facemasks are used to protect those occupationally exposed to, or at increased risk of, severe illness caused by influenza or other respiratory diseases [9–12]. Although the filters have been suggested to trap airborne particles very efficiently, filtration efficiencies have not been tested in real-world environments and may not be as high as suggested when trapping small particles in ambient air [13, 14].

Although N95 facemasks are commonly used, reports on their protective effects are limited. To the best of our knowledge, only three single-blind studies have been conducted, which reported that use of a typical N95 facemask reduced blood pressure and heart rate variability in healthy volunteers and patients with coronary heart disease, respectively [15–17]. A chamber study using N95 facemasks to control PM_2.5_ exposure indicated that, in the absence of filtration, PM exposure impaired vasomotor function and increased heart rate variability in overweight middle-aged and elderly adults [18]. More field experiments are required to measure the actual protective effects of facemasks. We hypothesize that changes in levels of subclinical biomarkers caused by exposure to PM in ambient particles can be influenced by wearing a N95 facemask. Therefore, we conducted a double-blind, randomized controlled trial to investigate the effects of N95 facemasks on human respiratory inflammation, endothelial dysfunction, and oxidative stress on days of severe air pollution.

## Methods

### Study participants

We calculated that a sample size of 14 was required to detect the minimal expected changes in urinary creatinine-corrected malondialdehyde levels before and after exposure of subjects wearing authentic and sham facemasks to air pollution (See Additional file 1 for more details). Hence, we recruited 15 healthy volunteers from Peking University (PKU); their characteristics are listed in Table 1. All were nonsmokers who were not on regular medication, had no history of coronary or respiratory disease, and no symptoms of upper airway infection commencing 4 weeks prior to study initiation until all measurements had been completed. Groups of four (or, in one instance, three) lived in the same dormitories, and the groups were required to spend the days immediately preceding the experimental days, and the experimental days, together, maintaining the same time schedules, activities, and diets. The study was approved by the Institutional Review Board of the PKU Health Science Center (approval no. 13073), and written informed consent was obtained from all participants before enrollment.

**Table 1:** Characteristics of the 15 study participants

Parameter	Mean (±standard deviation)
Age (years)	20.0 (±1.0)
Weight (kg)	56.2 (±7.0)
Height (cm)	169.0 (±7.0)
Body mass index (kg/m^2^)	19.1 (±2.0)
Sex (male/female)	7/8

### The double-blind, randomized, controlled crossover study design

**Fig. 1:**
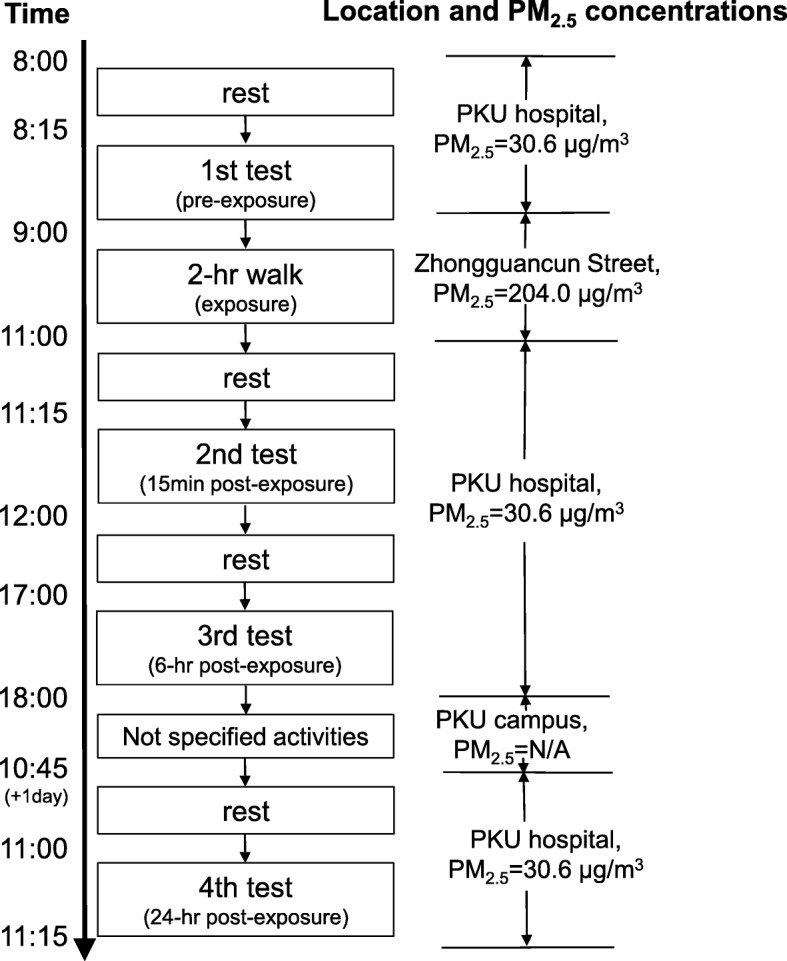
Timetable of the subjects and the average PM_2.5_ concentration during the visits

We performed a double-blind, randomized, controlled crossover study on two days associated with severe air pollution in Beijing, China (daily average PM_2.5_ concentration = 246.1 μg/m^3^ and 258.0 μg/m^3^, respectively) from November 2013 to February 2014. On the first clinical visit, each subject underwent tests including biomarker measurements and collection of samples for further biomarker analyses, according to the timetable shown in Fig. 1. These tests were performed in the basement of the PKU hospital, where the average PM_2.5_ concentration was 30.6 ± 3.1 μg/m^3^ over the entire study period (an air filtration system was operative). Next, each participant was randomized to wear a reusable facemask with or without a high-efficiency filter. Wearing the facemasks, the subjects were asked to walk together along a defined (off-campus) route with busy traffic for 2 h. When they returned to the hospital, they were immediately told to remove the facemasks and have a rest, and the second and third tests were performed after 15 min and 6 h of rest. The fourth test was performed at 11 a.m. on the next day, 24 h after the 2 h walk. After a 1-month washout interval, the subjects who wore real and sham facemasks exchanged roles and the entire procedure was repeated. All the subjects were required to complete the two visits at the same time.

### Air pollution monitoring

During the two clinical visits, the levels of air pollution and meteorological parameters were collected from the Peking University Urban Atmosphere Environment Monitoring Station (PKUERS), which located on the roof of a six-story building (~ 20 m above the ground). The station has been used for monitoring ambient pollutants in numerous studies [19–21] and can represent the air pollutants at a relative large scale including the walking route in our study. The station provided 1-min PM_2.5_ (Model 1400 TEOM; Thermo), sulfur dioxide (SO_2_) (Model 43i-TL; Thermo), nitrogen oxides (NO_x_) (Model 42i-TL; Thermo), ozone (O_3_) (Model 49i; Thermo), carbon monoxide (CO) (Model 48i-TLE; Thermo), temperature and relative humidity (Met One Instruments Inc., Grants Pass, OR, USA). Especially, to better assess the PM exposure during the two 2-h walks, real-time PM_2.5_ concentrations were measured at 1-min intervals using a Grimm optical particle counter (model 1.108; Grimm, Germany), carried in the backpack of a subject (data shown in Table 2).

**Table 2:** Mean Concentrations of pollutants during the 2-h walk in the two visits

Pollutants	Concentration (Min-Max, SD)
PM_2.5_, μg/m^3^	204.0 (101.0–326.6, 58.5)
CO, ppm	3.6 (1.5–6.1, 1.3)
O_3_, ppb	5.4 (0.1–36.1, 8.2)
SO_2_, ppb	37.2 (14.0–69.0, 15.1)
NO, ppb	65.5 (30.0–290.0, 51.2)
NO_x_, ppb	122 (46.5–563.5, 57.4)
Temperature, °C	0.3 (−2–3,1.56)
Relative Humidity,%	58.3 (38–81,16.6)

*PM2.5* particulate matter with aerodynamic diameter less than 2.5 μm, *CO* carbon monoxide, *O3* ozone, *SO2* sulfur dioxide, *NO* nitric oxide, *NOx* nitrogen oxides

### Facemask filtration efficiencies

We tested the filtration efficiencies of the six most popular types of N95 facemasks available from the two largest Chinese e-commerce websites (Taobao.com and Tmall.com), which were visited by approximately 25% of all Chinese shoppers in 2013 [22]. All facemasks were certified by the National Institute for Occupational Safety and Health (NIOSH) under regulation 42 CFR 84 [23], and their filtration efficiencies were evaluated using identical NIOSH procedures by Beijing Municipal Institute of Labor Protection, China (See details in the Additional file 1).

**Fig. 2:**
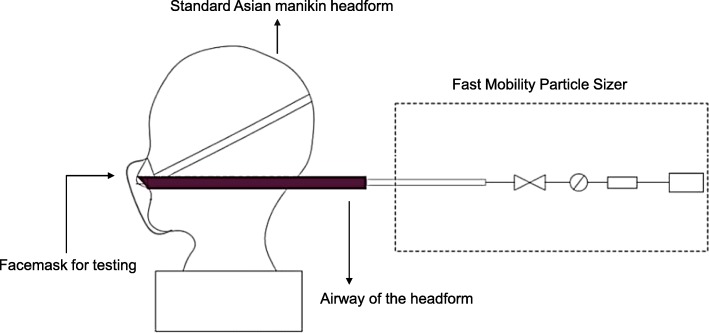
Experimental setup for measuring filter efficiencies of N95 facemasks with the headform based on particle number in ambient air. Key facial parameters of a medium-size headform in China include face length (122 mm), face width (145 mm), and interpupillary distance (62.5 mm) [24]

A manikin headform based on standard Asian faces was used to test the filtration efficiencies of particles in ambient air [24]. Compared with a human participant, the headform was more representative and could avoid interference of exhaled breath. A Fast Mobility Particle Sizer (model 3091; TSI Inc., USA) was used to measure the number concentrations of particles of diameter 5.6–560 nm in 32 size channels at a flow rate of 10 L/min. The experimental setup is presented in Fig. 2. The most effective type of the six facemasks (Respirator 3200 with Particulate Filter 3701CN; 3 M, USA) were used in the study. All the filtration efficiencies in this study were evaluated based on particle number concentration.

To ensure that the study was double-blinded, we used reusable facemasks without filters (Respirator 3200; 3 M, USA) as sham devices, which could not remove any particles, to control for potential placebo effects. Before the visits, all the participants were trained on how to wear and remove the facemasks correctly. Subjects still encountered respiration resistance even when the facemasks contained no filters; they could not tell whether they were wearing real or sham facemasks, which was confirmed in every participant.

### Biomarker measurements

#### Sample collection

Exhaled nitric oxide (eNO) and the levels of six pro-inflammatory cytokines (interleukin [IL]-1α, IL-1β, IL-2, IL-6, IL-8, and tumor necrosis factor [TNF]-α) in exhaled breath condensate (EBC) were measured to evaluate airway inflammation [25–27]; urinary creatinine-corrected malondialdehyde (MDA) levels were measured to quantify systemic oxidative stress [28, 29]; and pulse wave analysis (PWA) (including assessment of augmentation pressure [AP], the augmentation index [AIx], and ejection duration [ED]) was performed to evaluate arterial stiffness (a measure of endothelial dysfunction) [30–32]. The eNO and cytokine levels were measured, and PWA performed, at every test, but MDA measurements were obtained at only the pre-exposure (first) and the 24-h post-exposure (fourth) tests associated with each experiment.

#### Exhaled nitric oxide (eNO)

Exhaled air samples were collected following the instruction of ATS/ERS 2005 [33]. Before sampling, subjects must gargle for 3 times and wear a nose clip. Then they were asked to inhale deeply, and then exhale to wash the “dead space” from the device for 3 times. Subjects were instructed to inhale to tidal capacity and then exhale into a 4-L Teflon bag with a flow of 150 L/h and a pressure of 13 cm H_2_O (to close the soft palate and prevent nasal exhalation). A tube filled with activated carbon was used to filter out NO from the ambient air and was shown to remove up to 99.6% of NO in a laboratory simulation of the protocol used in the field. The device was equipped with a flow meter as a flow restrictor and a pressure indicator. Concentration levels of eNO in the samples were determined with a NO–NO_2_–NO_x_ analyzer (model 42i; Thermo, USA). We recorded 10 readings after the concentration showed in the instrument is stable and used their average as the final result. This method has been used in our previous studies [25, 34, 35].

#### Cytokines in exhaled breath condensate (EBC)

Exhaled breath condensate (EBC) was collected by EcoScreen (Jaeger, Germany), and the procedure of collection and storage followed the recommended instruction [36]. Before collection, subjects were required to rest for at least 5 min, gargle for 3 times and wear a nose clip, sequentially. For collection, the subjects breathed normally for 5 min, and the samples collected were stored in − 80 °C.

The BD™ Cytometric Bead Array (CBA) method was performed to determine the cytokine concentrations in EBC with a multiplex fluorescent bead immunoassay (Cytometric Bead Array, BD-Biosciences, San Jose, CA, USA). Cytokines in test samples and recombinant standards as bound to capture beads were detected by PE-conjugated detection antibodies and measured by flow cytometry (FACS Calibur, BD-Biosciences, San Jose, CA, USA; [37]). Following acquisition of sample data, the sample results were generated in graphical and tabular format using the CBA analysis software (BD-Biosciences, San Jose, CA, USA). The broad dynamic range of fluorescence detection via flow cytometry and the efficient capturing of multiple analytes via suspended particles enable the CBA system to obtain the concentration of an unknown in substantially less time and using fewer sample dilutions compared to the conventional ELISA methodology, and the CBA method has been widely used in serum cytokine detection [38–40]. To avoid the potential influence from pH in EBC (pH = 5.6–6.8), 10 μl phosphate buffer saline (PBS) was added into every 40 μl EBC to modify the pH of the samples to 7.4. Detection limits of the six proinflammatory cytokines (IL-1α, IL-1β, IL-2, IL-6, IL-8, TNF-α) are 2.0, 2.4, 2.6, 2.4, 2.4, 3.8 pg/mL, respectively.

#### Urinary malondialdehyde and creatinine

As the product of lipid oxidation, urinary creatinine-corrected-malondialdehyde presents the systemic oxidative stress in a relatively long term. We thus only determined it at pre-exposure (baseline) and 24-h post- exposure, and morning urine samples were required for collection. Malondialdehyde, released from its bound form(s) in urine by acid treatment, was measured as thiobarbituric acid derivative, using HPLC-UV (Model e2695, Waters, USA) according to [41]. Briefly, 150 μL urine, 450 μL thiobarbituric acid (TBA) solutions (Sigma-Aldrich, St. Louis, USA), and 900 μL 0.5 mol/L phosphorous acid were added into 1.5 mL plastic centrifuge tube. The mixtures were incubated at 95 °C for 1 h, cooled in ice water for 5 min, followed by 5-min centrifugation (5000 g/min). HPLC with chromatographic column X-Terra RP18 C18 (X-Terra, USA) was used to detect maloniadehyde concentration in the mixtures at the wavelength of 532 nm by Photodiode Array (PDA) detector (Waters 2998, USA) with the flow rate of 0.8 mL/min. The mobile phase was phosphate buffer (pH = 6.8) and methanol (60:40, *V*/V).

In our study, concentrations of maloniadehyde were corrected by creatinine due to its highly potential influence by metabolism. Urinary creatinine levels were analyzed by a commercial ELISA kit (Jiancheng Bioengineering Institute, Nanjing, China), based on the Jaffe reaction and spectrophotometry [42].

#### Pulse wave analysis (PWA)

Pulse wave analysis was performed using micromanometer applanation tonometry (Millar Instruments, Texas, USA) of the radial artery at the wrist. The pulse wave data was directly collected into a portable computer and an averaged waveform was generated by the SphygmoCor™ system (AtCor Medical, Sydney, Australia) in accordance with the manufacturer’s recommendations. Briefly, pulse wave analysis derives an aortic pulse pressure waveform from the radial artery wave via a mathematical transfer function. The arterial pressure waveform is a composite of the forward pressure wave created by ventricular contraction and a reflected wave generated by peripheral vascular resistance. The systolic part of central arterial waveform is characterized by two pressure peaks. The first peak is caused by left ventricular ejection, whereas the second peak is a result of wave reflection. The difference between both pressure peaks reflects the degree to which central arterial pressure is augmented by wave reflection. Augmentation pressure (mm Hg), is the difference between the second and first systolic peaks. Augmentation index (%) is defined as the percentage of the central pulse pressure which is attributed to the reflected pulse wave and, therefore, reflects the degree to which central arterial pressure is augmented by wave reflection. Ejection Duration (second) is defined as the sum of time to wave reflection (Tr) and systolic duration of reflection wave (SDR) [43–45].

#### Statistical analyses

All biomarker levels were subjected to analysis of skewness and kurtosis to explore whether they exhibited Gaussian distributions (α = 0.05). The paired Student’s *t*-test was used to compare biomarker levels before and after the experiments. To control the family-wise type I error rate at a 0.05 level, a Bonferroni correction was applied; with 11 biomarkers measured in this study, each individual 2-sided test was considered statistically significant relative to a 0.005 significance level. All analyses were performed with the aid of R statistical software (version 2.15.3, R Core Team 2013).

## Results

### Filtration efficiencies

**Fig. 3:**
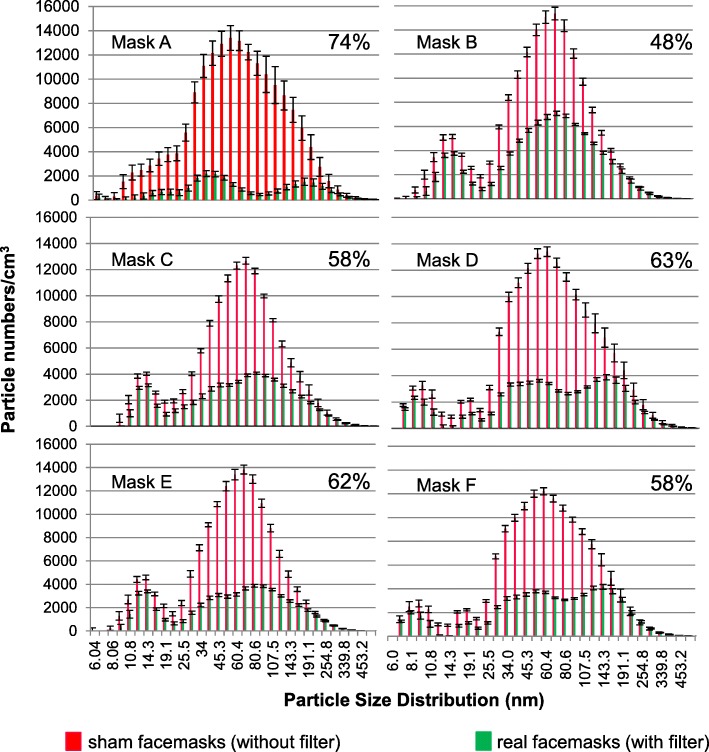
Filtration efficiency of 5.6–560 nm particles in ambient air of six N95 facemask types. Here the filtration efficiency is defined as proportion of particles that cannot go through the filters. The total efficiency of each N95 facemask for particles between 5.6 and 560 nm is listed on the top right corner of each graph. $\mathrm{Efficiency}=\left(1-\frac{particle\kern0.17em numbers\kern0.17em for\kern0.17em facemask\kern0.17em with\kern0.17em filter}{particle\kern0.17em numbers\kern0.17em for\kern0.17em facemask\kern0.17em with out\kern0.17em filter}\right)\times 100\%$

In tests following the NIOSH guidelines, we found that all six types of facemask met the standard N95 requirements with efficiencies of 97.2–99.4% (detailed data shown in Additional file 1). However, the performance of the facemasks in terms of filtration of ambient PM 5.6–560 nm in diameter was less impressive. The most effective facemask removed only 75% of the particles and the least effective one 48% (Fig. 3). Although filtration efficiency varied greatly at different particle diameters, particles removed most efficiently by all the six facemasks was in the range of 69.8–93.1 nm in diameter.

### Pollution descriptive statistics

Additional file 1: Table S1 lists the average air pollutant concentrations during the 2-h walks of the two experiments; the PM_2.5_ concentrations were above 101 μg/m^3^ (average, 204 μg/m^3^), consistent with those of typical winter air pollution episodes in Beijing [6, 46].

### Biomarker descriptive statistics

**Fig. 4:**
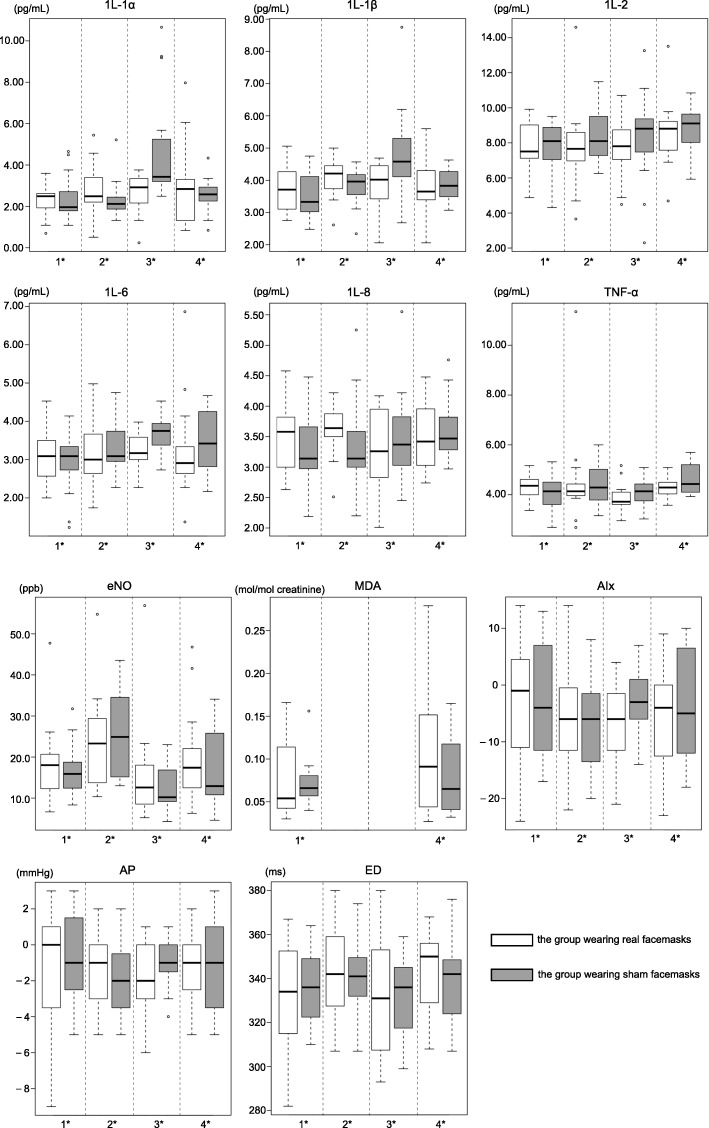
Biomarker concentrations of the subjects in 4 tests. 1*: pre-exposure; 2*: 15-min post-exposure; 3*: 6-h post-exposure; 4*: 24-h post-exposure

Figure 4 shows the biomarker levels of the four tests. The levels of all six cytokines (IL-1α, IL-1β, IL-2, IL-6, IL-8, and TNF-α) in EBC were above the analytical detection limits (compared with negative controls). In the first test (before the walk), no significant difference in any biomarker level was evident between the groups wearing real and sham facemasks during the walk.

### Effects of facemasks on airway inflammation

**Fig. 5:**
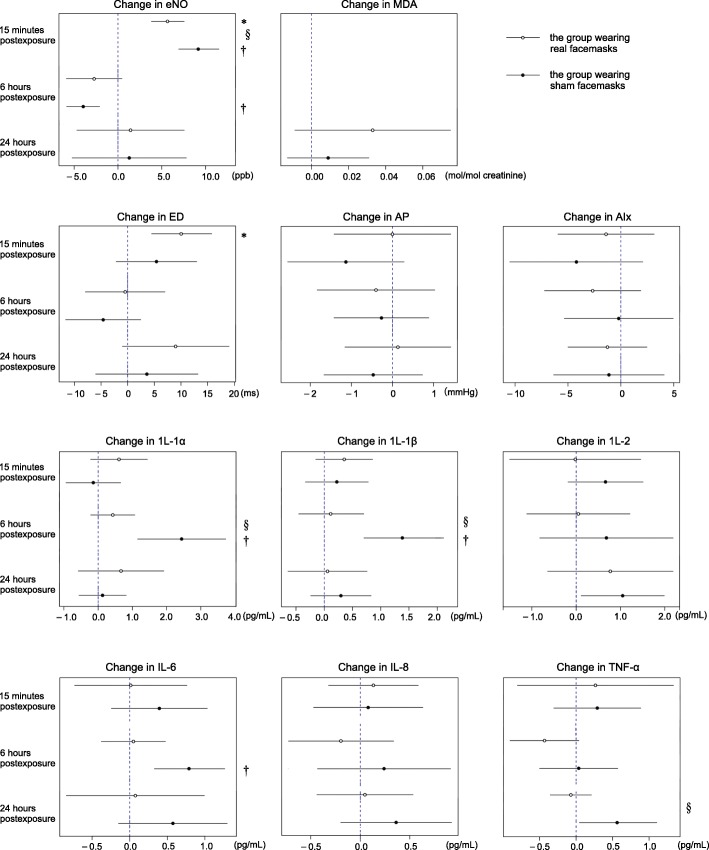
Mean changes of biomarkers (95% CI) compared to the pre-exposure (1st) test in the subjects. * Significantly different from baseline in the group wearing real facemasks, *p* < 0.005 † Significantly different from baseline in the group wearing sham facemasks, *p* < 0.005. §Significantly different between the groups wearing real and sham facemasks in the test, *p* < 0.005.

After the 2-h walk, the levels of all biomarkers changed (Fig. 5). At 15 min post-exposure, the concentration of eNO in both groups increased significantly (*p* < 0.005), but the increase in the group wearing real facemasks was 38.3% less than the sham group (*p* < 0.005). At 6 and 24 h post-exposure, the eNO concentrations returned to relatively low levels, and did not differ significantly between the groups who had worn real and sham facemasks (*p* > 0.05).

In terms of cytokine levels in EBC, we found no significant change at 15 min, 6 h, or 24 h post-exposure in the group that had worn real facemasks (*p* > 0.05) (Fig. 5). In the sham group, the levels of IL-1α, IL-1β, and IL-6 in EBC were significantly elevated at 6 h post-exposure (all *p* < 0.005). Compared to the group wearing real facemasks, the IL-1α and IL-1β levels in EBC at 6 h post-exposure were significantly higher (82.4 and 91.4%, respectively) in the sham group (both *p* < 0.005). Similarly, the TNF-α level at 24 h post-exposure was significantly higher in the sham group (by 112.5%) After adjustments for multiple comparisons, changes in the other outcomes were not statistically significant (Fig. 5).

### Effects of facemasks on oxidative stress and endothelial dysfunction

As shown in Fig. 5, at 24 h post-exposure, the urinary creatinine-corrected MDA concentrations were higher (compared with baseline) in both groups, but did not differ significantly between the groups (*p* > 0.05). Of the endothelial dysfunction biomarkers, only the ED increased significantly (compared with baseline) in the group wearing real facemasks, at 15 min post-exposure (*p* < 0.005). The level was 40.0% higher than that of the sham group, but the difference was not significant (*p* > 0.05).

## Discussion

Using a robust, double-blind, randomized, controlled crossover design, we assessed the effects of wearing N95 facemasks on days of severe air pollution in Beijing, China. We measured changes in the levels of biomarkers reflecting airway inflammation, oxidative stress, and endothelial function four times within 24 h after a 2-h exposure to ambient air (walking along a busy road). We evaluated six of the most popular types of N95 facemasks in terms of ambient air filtration efficiency during typical episodes of Beijing pollution [46]. Our study recruited young healthy adults and observed the biomarkers changes linking interventions in air pollution, which might be particularly important because they are less sensitive to air pollution exposure than other subpopulations [47]. To minimize subjective differences, the characteristics of all volunteers were similar; and they maintained identical time schedules and dietary habits before and during the study days, reducing exposure differences and minimizing individual bias. Moreover, we gave facemasks without filters to the sham group, to reduce any possible subjective bias and maintain the double-blind design. Previous studies did not feature sham facemasks, and the outcomes may thus have been influenced by anxiety [15–17].

To the best of our knowledge, this is the first study to describe the real-world PM filtration capacities of available facemasks in terms of particle size fractions between 5.6 and 560 nm in diameter (Fig. 3). Previous laboratory studies on filtration performance suggested that such facemasks were very efficient. However, these experiments used particles of specific composition (e.g., sodium chloride) [48–50]. Only one study [15] reported the filtration efficiency of diesel exhaust, but did not distinguish between the different size fractions. The reduced facemask filtration efficiencies of ambient air may be explained by the fact that N95 facemasks were initially designed and are commonly used for occupational protection in industrial and healthcare settings [51]. The filtration efficiencies of specific particles cannot be generalized to ambient particles, first because chemical species vary greatly in penetration, and, second, because ambient PM is a very complicated mixture exhibiting large spatial and temporal variations [13, 52]. Previous laboratory studies indicated that the filtration efficiencies were influenced by multiple factors, including particle sizes, particle compositions, aerosol concentrations, etc. [53–56]. Evaluation of facemask filtration efficiencies in terms of different PM size fractions is important. It has been suggested that the association between health effects and number concentration of fine particulates depended on particle size, and ultrafine particles are primarily responsible for the adverse health effects [27, 38–41]. Smaller particles would have greater toxicity than larger particles due to their larger active surface area absorbing more toxic chemicals and higher deposition efficiency in the respiratory tract [35, 57–60]; besides, number concentration of ultrafine particles is much higher, which constitutes over 90% of aerosol fine particles in China [36]. Fibrous facemask filters are least effective when removing particles ranging from 0.1 to 0.4 μm in diameter [54, 61]. Thus, N95 facemasks might not exhibit filtration efficiencies as impressive as expected.

Many studies have confirmed that inflammation is one of the most critical biological pathways affected by PM [62, 63]. The eNO level is an acute clinical surrogate of airway inflammation, and increases following exposure to PM [25, 27, 64, 65]. We found a significant acute increase in the eNO concentration following high-level exposure to PM in subjects wearing real or sham facemasks, but the increase in the group wearing real facemasks was substantially less than that in the sham group. This suggests that N95 facemasks may reduce, but not eliminate, respiratory inflammation induced by high-level PM exposure. Given the features of facemask efficiency described above, the fact that the eNO level increased in the group wearing real facemasks implies that smaller particles contributed significantly to inflammation. However, the possibility that high-level gaseous pollutants (e.g., nitrogen oxides and sulfur dioxide) were responsible for the elevation in eNO [66, 67] cannot be excluded. Additional research is required.

Many reports have shown that short-term exposure to PM increased the levels of inflammatory cytokines including IL-6, IL-1β, TNF-α, IL-γ, and IL-8, in bronchial fluid, EBC, and sometimes blood [68–73]. Although the sources of cytokines and the extent of their involvement in the systemic inflammatory response following PM exposure remain unclear, pulmonary inflammation may be attributable to both innate immune cells (neutrophils and macrophages) and adaptive immune cells such as T cells [71]. Previous studies suggested that PM inhalation induced inflammatory cytokine release by neutrophils and macrophages 6–24 h later [74–77]. We found that the levels of TNF-α and IL-6 in EBC in the group wearing sham facemasks increased significantly 6 and 24 h after exposure, respectively. However, PM-induced T cells have been suggested to contribute to the observed increases in IL-1, IL-4, IL-6, and IL-10 [70]. We found that IL-1α, IL-1β, and IL-6 levels in EBC increased significantly 6 h after exposure in the group wearing sham facemasks. In contrast, cytokine levels in EBC did not change significantly following PM exposure in the group wearing real facemasks. Indeed, the IL-1α and IL-1β levels in EBC 6 h after exposure were significantly lower than in the sham group. Changes in the cytokine levels paralleled those of eNO, suggesting that, upon exposure to PM, the increase in pulmonary inflammation induced by either immune cells or T cells was effectively prevented with the use of a facemask.

Applanation tonometry of the radial artery and PWA are typically used to measure arterial stiffness and central aortic blood pressure. Controlled experiments have shown that short-term exposure to ambient PM may trigger acute vasoconstriction, vascular endothelial dysfunction, and increases in blood pressure and arterial stiffness, which may in turn explain the observed increase in associated cardiovascular events [32]. PWA accurately records peripheral pressure waveforms, and generates the corresponding central waveform, from which the AIx, AP, and ED can be derived [78]. However, the effects of air pollution on PWA outcomes have received little attention. It has been reported that exposure to wood and tobacco smoke, as well as diesel exhaust, increased arterial stiffness [32, 71, 79]. We found no significant change in the AIx or AP following PM exposure. The ED was significantly increased in the group wearing real facemasks 15 min after exposure, suggesting that acute PM exposure increased arterial stiffness in those wearing facemasks. The increase was not significant in the sham group. ED is a complex surrogate of cardiovascular effects, and may become elevated when the heart rate changes [80]. Although the volunteers tolerated the facemasks well, the masks increased respiration resistance, perhaps raising the heart rate [12, 15, 81] and thus the ED. Another possibility is that the wearing of a facemask may trigger deeper breathing, increasing the inhalation flow rate. Thus, finer particles may deposit in deeper sections of the respiratory system, causing more severe cardiovascular injury. This hypothesis could also explain the decreases in AP and AIx, and the increase in MDA, in the group wearing real facemasks, although the changes did not differ significantly from those in the sham group. The mild changes in AP and AIx are possibly attributable to the fact that we studied young healthy volunteers, whose cardiovascular systems were less vulnerable to PM exposure. N95 facemasks exhibit respiration resistance that may stimulate the cardiovascular system, and the U.S. FDA does not recommend their use by children, older subjects, or those with chronic respiratory or cardiac conditions, unless their healthcare providers indicate that the masks are appropriate [82]. Additionally, we found no significant difference in the urinary MDA level (a surrogate marker of systemic oxidative stress) before and after exposure, possibly because the MDA concentration typically peaks 40–70 h after exposure [29], or because our volunteers were not susceptible to such stress. Further work is required.

We measured biomarker levels three times following PM exposure to increase our understanding of the mechanisms and timing of the respiratory, pulmonary, and systemic inflammatory responses to sustained exposure, particularly PM_2.5_, in healthy subjects. Moreover, our findings have significant implications for public health. First, the fact that the filtration efficiency of ultrafine ambient particles was low indicates that facemasks that filter smaller particles better are required. Second, exposure to ambient air pollution was associated with direct, measurable adverse effects even in young healthy adults habitually exposed to such pollution. Third, N95 facemasks appeared to effectively reduce short-term PM-induced respiratory inflammation, but are unlikely to completely eliminate inflammation. Fourth, we did not observe significant cardiovascular benefits produced by N95 facemasks. Although more work is needed to confirm or refute, healthcare providers should be cautious when recommending N95 facemasks to susceptible subjects.

Our study had certain limitations. First, our sample size was small, which may have masked the significance of changes in the levels of some biomarkers. Additional tests with larger numbers of subjects are necessary. Second, our experimental days featured not only high-level PM, but also gaseous pollutants, which may have influenced our findings. Third, although the two air pollution episodes studied were similar, the specific concentrations of various pollutants may have varied, affecting our results and conclusions. Fourth, we did not consider potential face-seal leaks of the facemasks, which can be a penetration pathway for aerosol particles. Fifth, we recruited young healthy adults rather than subjects more susceptible to air pollution, and the changes in some biomarker levels might thus have been minimal in our volunteers. Although it is likely that our findings will be relevant in other populations, further studies are required.

## Conclusions

In summary, acute respiratory inflammation in healthy young adults caused by air pollution was partially reduced by N95 facemasks, but systemic oxidative stress and endothelial dysfunction may not have been alleviated. Although the clinical significance of these findings long-term remains to be determined, this study provides quasi-experimental, mechanistic data for the potential cardiopulmonary effects associated with wearing facemasks during episodes of pollution.

## Additional file

Additional file 1: **Table S1.** Filtration Efficiencies and resistance of six types of facemasks. (DOCX 36 kb)

## Abbreviations

AIx: augmentation index
AP: augmentation pressure
CBA: Cytometric Bead Array
EBC: exhaled breath condensate
ED: ejection duration
ELISA: enzyme-linked immunosorbent assay
eNO: Exhaled nitric oxide
FDA: Food and Drug Administration
h: hour
IL: interleukin
MDA: malondialdehyde
min: minute
NIOSH: National Institute for Occupational Safety and Health
PKU: Peking University
PKUERS: Peking University Urban Atmosphere Environment Monitoring Station
PM: particulate matter
PM_2.5_: particulate matter of aerodynamic diameter less than 2.5 μm
PWA: pulse wave analysis
TNF: tumor necrosis factor
