# Supplementary material for: The effects of facemasks on airway inflammation and endothelial dysfunction in healthy young adults: a double-blind, randomized, controlled crossover study
Source: Part Fibre Toxicol. 2018 Jul 4;15:30. doi: 10.1186/s12989-018-0266-0 (PMC6032602; doi:10.1186/s12989-018-0266-0)
Supplement: Supplementary file 1 — Table S1. Filtration Efficiencies and resistance of six types of facemasks. (DOCX 36 kb) [file 12989_2018_266_MOESM1_ESM.docx]

**Additional file**

**The effects of facemasks on airway inflammation and endothelial dysfunction in healthy young adults: a double-blind, randomized, controlled crossover study**

Tianjia Guan, Songhe Hu, Yiqun Han, Ruoyu Wang, Qindan Zhu, Yaoqian Hu, Hanqing Fan, Tong Zhu

**Sample Size Calculation**

Based on our previous studies, urinary creatinine-corrected malondialdehyde (MDA) was supposed to be less sensitive than other measured biomarkers, thus it was used in sample size calculation in this study [S1-S3]. The largest range of urinary malondialdehyde was found to be 0.10 mmol/mol creatinine (95% CI: 0.077, 0.124) with a standard deviation of 0.012 mmol/mol. Compared to the lower PM_2.5_ exposure group (< 61.8 μg/m^3^), MDA estimates in higher exposure to PM_2.5_ (165- 248.2 μg/m^3^) were higher (mean odds ratio = 1.19).

If we assumed the difference in PM_2.5_ exposure level was 200 μg/m^3^, the difference between the null hypothesis mean and the alternative hypothesis mean was set as 3.8, and the standard deviation was estimated as 2.4. To adjust multiple comparisons, we set the significance level as *p*= 0.0005. We selected relatively conservative data here to strengthen the estimation.

Therefore, a sample size of 14 achieves 85% power to detect a difference of -3.8 between the null hypothesis mean and the alternative hypothesis mean with an estimated standard deviation of 2.4 and with a significance level (alpha) of 0.00050 using a two-sided one-sample t-test [S4, S5]. The sample size calculation was accomplished with PASS (version 11.0, NCSS Inc., USA).

**42 CFR part 84, National Institute for Occupational Safety and Health (NIOSH) certification**

The 42 CFR part 84, National Institute for Occupational Safety and Health (NIOSH) certification tests use the most penetrating aerosol size, 0.3 µm aerodynamic mass median diameter, of either a mildly degrading particulate such as sodium chloride (NaCl) for N- series respirators, or a highly degrading oil such as Dioctyl Phthalate (DOP) for R-series respirators.

In this study, charge sodium chloride (NaCl) aerosols (count median diameter: 0.075 ± 0.02 μm) were used to measure filter penetration levels of air-purifying particulate respirators photometrically using TSI 8130 Automated Filter Tester (TSI 8130, Inc., Shoreview, Minn.) at a flow rate of (85 ± 4) L/min in an environment of 30 (± 10) % relative humidity at 25(± 5) ° C. TSI model 8130 is designed for and has been widely used in NIOSH particulate respirator certification [S6, S7]. This testing was accomplished by Beijing Municipal Institute of Labor Protection, China.

**Supplemental Table 1. Filtration Efficiencies and resistance of six types of facemasks**

Abbreviations: NIOSH= [National Institute for Occupational Safety and Health](https://www.cdc.gov/NIOSH/)

| Type of Facemasks | Parameters tested following the 42 CFR part 84, NIOSH guideline | | Filtration efficiency of PM_2.5_ number concentration in ambient air |
| --- | --- | --- | --- |
|  | Filtration efficiency | Resistance (mmH_2_O) |  |
| Mask A | 99.453% | 30.5* | 92.19% |
| Mask B | 97.180% | 8.2 | 58.73% |
| Mask C | 99.453% | 9.2 | 70.19% |
| Mask D | 99.296% | 14.3 | 70.56% |
| Mask E | 99.388% | 9.0 | 78.24% |
| Mask F | 99.281% | 7.7 | 67.24% |

* The resistance of filter media (3M-3710CN), not the whole mask.

**Supplementary References**

S1. Lin W, Huang W, Zhu T, et al. Acute respiratory inflammation in children and black carbon in ambient air before and during the 2008 Beijing Olympics. Environmental Health Perspectives 2011;119(10):1507.

S2. Lin W, Zhu T, Xue T, et al. Association between changes in exposure to air pollution and biomarkers of oxidative stress in children before and during the Beijing Olympics. American Journal of Epidemiology 2015;181(8):575-583.

S3. Han Y, Zhu T, Guan T, et al. Association between size-segregated particles in ambient air and acute respiratory inflammation. Science of the Total Environment 2016;565:412-419.

S4. Machin, D., Campbell, M., Fayers, P., and Pinol, A. Sample Size Tables for Clinical Studies, 2nd Edition. Blackwell Science 1997. Malden, MA.

S5. Zar, Jerrold H. Biostatistical Analysis (Second Edition). Prentice-Hall 1984. Englewood Cliffs, New Jersey.

S6. Rengasamy S, Miller A, Eimer BC. Evaluation of the Filtration Performance of NIOSH-Approved N95 Filtering Facepiece Respirators by Photometric and Number-Based Test Methods. Journal of Occupational & Environmental Hygiene 2011;8(1):23-30.

S7. Rengasamy S, Shaffer R, Williams B, et al. A comparison of facemask and respirator filtration test methods. Journal of Occupational & Environmental Hygiene 2016;14(2):92-103.
